# A dataset on odor intensity and odor pleasantness of 222 binary mixtures of 72 key food odorants rated by a sensory panel of 30 trained assessors

**DOI:** 10.1016/j.dib.2021.107143

**Published:** 2021-05-15

**Authors:** Yue Ma, Ke Tang, Yan Xu, Thierry Thomas-Danguin

**Affiliations:** a State Key Laboratory of Food Science and Technology, Jiangnan University, 1800 Lihu Avenue, Wuxi, Jiangsu 214122, China; b Key Laboratory of Industrial Biotechnology of Ministry of Education, Jiangnan University, 1800 Lihu Avenue, Wuxi, Jiangsu 214122, P. R. China; c Centre des Sciences du Goût et de l'Alimentation, INRAE, CNRS, AgroSup Dijon, Université Bourgogne Franche-Comté, Dijon, France

**Keywords:** Odor interaction, Masking, Synergy, Partial addition, Dominance, Hedonic

## Abstract

This paper describes data collected on a set of 222 binary mixtures, based on a set of 72 odorants chiefly found in food, rated by 30 selected and trained assessors for odor intensity and pleasantness. The data included odor intensity (IAB) and pleasantness (PAB) of the mixtures, the intensity (IA, IB) and the pleasantness (PA, PB) of the two components. Moreover, the intensity (IAmix, IBmix) of the two components’ odor perceived within the mixture are included. The quality of the dataset was evaluated by checking subjects’ performance and by testing repeatability using the 24 duplicated trials for each attribute. This set of experimental data would be especially valuable to investigate theories of odor mixture perception in human and to test new models to predict odor perception of odor mixtures.

**Specifications Table**

**Table:** 

Subject	Food Science
Specific subject area	Sensory evaluation
Type of data	Microsoft Excel Worksheet containing 3 sheets: (1) odor information, (2) mean value after deleting sub47 (grouping and mean value of 222 trials after discarding one subject who was a systematic outlier sub.47), (3) individual data (raw individual psychophysical data points).
How data were acquired	Sensory evaluation: 222 sets of odor samples were evaluated by 30 selected and trained assessors from China.
Data format	Table in raw format (.xlsx)
Parameters for data collection	Variables included the intensity of odor A (IA) or odor B (IB), the intensity of odor A (IAmix) or odor B (IBmix) perceived within the mixture, the intensity of the binary mixture (IAB), the pleasantness of odor A (PA) or odor B (PB), the pleasantness of the binary mixture (PAB).
Description of data collection	The data presented in this study were collected from fifteen sessions across three months in Jiangnan University, Jiangsu, People's Republic of China. Before the formal experiment, two training sessions were conducted. In the formal sessions, a total of 222 trials, among which 24 were duplicated trials, were evaluated. Each session in the formal experiment comprised 14 to 15 trials, and each trial included three stimuli: two stimuli were single odorants, and the third stimulus was a binary mixture of these odorants. Each trial was presented to subjects in a random order, and one trial was evaluated by a maximum of 30 subjects.
Data source location	Institution: State Key Laboratory of Food Science and Technology, Jiangnan University City/Town/Region: 1800 Lihu Avenue, Wuxi, Jiangsu 214,122 Country: People's Republic of China
Data accessibility	Repository name: Portail Data INRAE Data identification number: 10.15454/51OVY6 Direct URL to data: https://doi.org/10.15454/51OVY6
Related research article	Ma, Y., Tang, K., Thomas-Danguin, T., & Xu, Y. (2020) [1]. Pleasantness of Binary Odor Mixtures: Rules and Prediction. Chemical Senses, 45(4), 303‑311. https://doi.org/10.1093/chemse/bjaa020 Ma, Y., Tang, K., Xu, Y., & Thomas-Danguin, T. (2021) [2]. Perceptual interactions among food odors: major influences on odor intensity evidenced with a set of 222 binary mixtures of key odorants. Food Chemistry (on line). https://doi.org/10.1016/j.foodchem.2021.129483

## Value of the Data

• Perceptual interactions in odor mixtures still constitute a major bottleneck to our understanding of the relationships between food volatile organic compounds (VOCs) and flavor perception. Only a few studies have focused on this topic, and most of them relied on small dataset because of the experimental difficulty to collect odor rating data with a significant panel of assessors. This dataset aimed to provide high quality data on intensity and pleasantness for a large set of binary odor mixtures.
• This large set of binary odor mixtures data can be useful for anyone who has an empirical interest in generalizing the rules and factors that are critical to the mixture-induced effects namely masking and synergy, and their consequence for food flavor and further aroma formulation.
• Everyday odors are the result of the perception of complex mixtures of odorants. This large dataset may be useful for anyone interested to understand the perception of real odors and their underlying mechanisms in human. For instance, the dataset can serve as benchmark to test models predicting the expected outcome after mixing odor-active compounds.

## 1 Data Description

The dataset is provided as an Excel file (.xlsx) and includes three sheets.

The first sheet “odor information” provides information about the 72 odorants used in the experiment. The sheet contains 7 columns: “CAS.” is the CAS number of the compound, “Odorant” is the common chemical name of the odorant, “Odor” indicates the main odor note of the compound, “cons. (mg/ml)” indicates the concentration of the odorant and “Solvent” the solvent in which the compound was diluted, “Purity” indicate the chemical purity of the odorant, “Trial number” indicates the identification number of the trials in which the compound was used.

The second sheet “mean value after deleting sub47” summarizes all the data for the average intensity and pleasantness of the 198 different binary odor mixtures, plus 24 duplicated binary odor mixtures, evaluated by all subjects except for subject 47 which was removed from the final data due to his bad performance 30 trained subjects. This sheet includes 14 columns: “Trial” is the identification number of the trial, i.e. the binary mixture, “R-Trial” indicates if the trial was duplicated and the identification number of the replicated trial, “Repeat” can be 1 or 2 and indicates which replicated trial it is, “Group” indicates which one of the 4 possible groups of trials the mixture belongs to (group E, comprising 50 trials, showed no significant difference in either intensity or pleasantness; group I, which included 52 trials, showed a significant difference in intensity only; group P, comprising 39 trials, showed a significant difference in pleasantness only; and group IP, comprising the remaining 57 trials, showed a significant difference in both intensity and pleasantness), “odor A” and “odor B” indicates the first and second odorant included in the binary mixture (trial). The last 8 columns report the mean sensory data for: “IA” mean odor intensity for odorant A out of mixture, “IAmix” mean odor intensity for odorant A in the mixture, “IB” mean odor intensity for odorant B out of mixture, “IBmix” mean odor intensity for odorant A in the mixture, “IAB” mean odor intensity for the whole mixture, “PA” mean odor pleasantness for odorant A, “PB” mean odor pleasantness for odorant B, “PAB” mean odor pleasantness for the binary mixture.

**Fig. 1:**
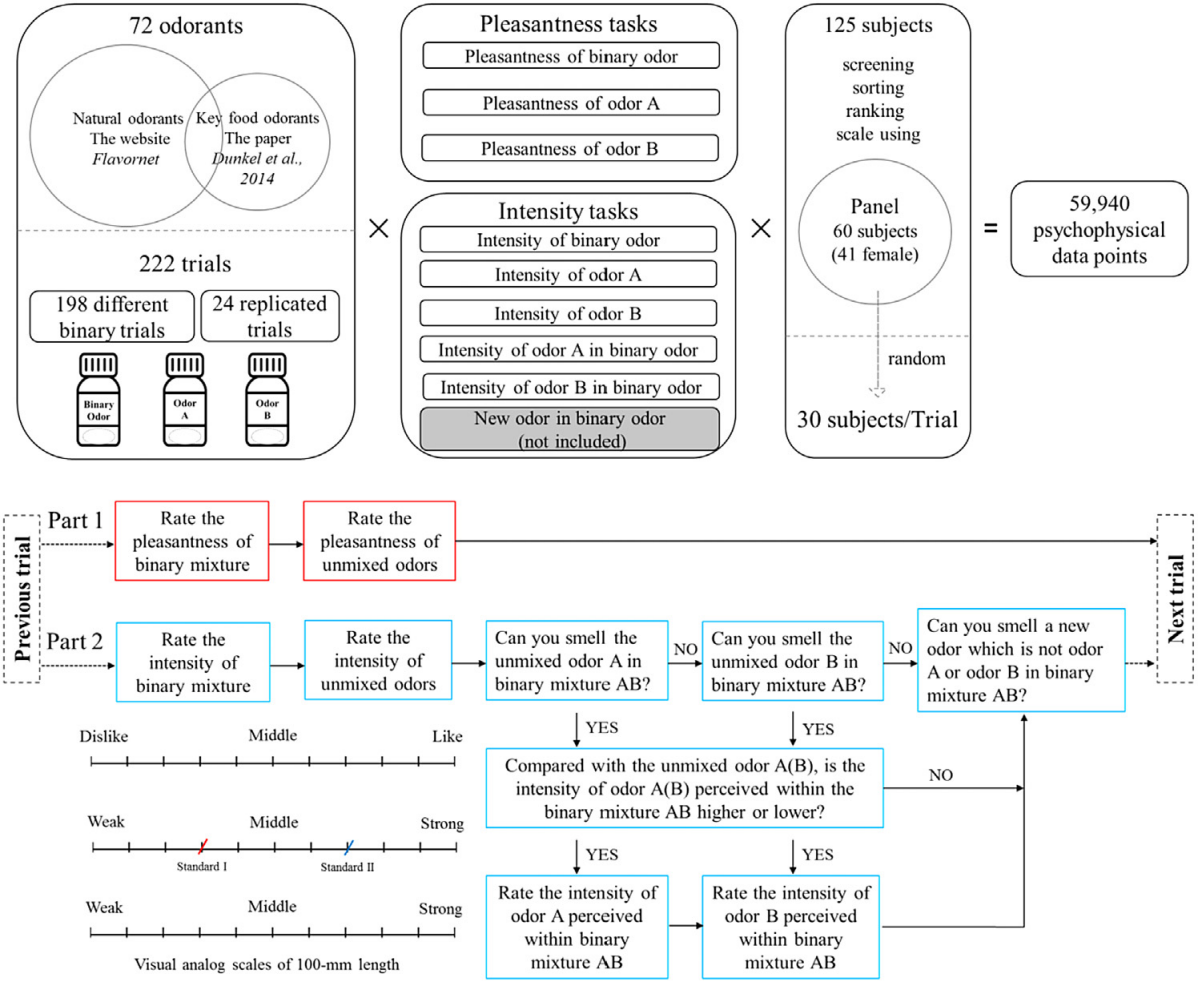
Schematic of psychophysical experiment data collection. The figure was modified from the related research articles ([1], 2021). The upper scale shown in the figure presented two ticks labeled as standard 1 and standard 2. This scale was only provided in an instruction sheet during the training session along with the standard samples. This instruction sheet was also provided at the beginning of the first two sessions to remind subjects with the intensity scale. However, for all the samples’ evaluation, we only used the bottom scale without the two ticks.

The third sheet “individual data” has 14 columns and gathers the raw data for the intensity and pleasantness of the 198 different binary odor mixtures, plus 24 duplicated binary odor mixtures, evaluated by 30 trained subjects. The sheet has 6660 lines of data that are the individual values collected for each odor sample for the variables “IA”, “IAmix”, “IB”, “IBmix”, “IAB”, “PA”, “PB”, “PAB” (columns 7 to 14), which represents a total of 53,280 individual psychophysical data points (in Fig. 1 we indicated a total of 59,940 psychophysical data points, but one variable (“new odor in binary odor”) was not included in the dataset). The first 6 columns in this sheet are respectively: “Sub” the identification number of a given subject, “Trial”, “R-Trial”, “Repeat”, “odor A” and “odor B” as previously detailed for the sheet “mean value after deleting sub47”.

## 2 Experimental Design, Materials and Methods

### 2.1 Subjects

One hundred twenty-five healthy subjects between the ages of 18 and 25 were recruited from Jiangnan University (China). Sixty-six of these subjects went through screening tests that evaluated their performance in discriminating between different odors qualities and different odor intensity levels, as well as their performance in logic scaling. To test their ability to evaluate odor quality and intensity, six samples comprising three different odorants at two concentration levels were provided. The subjects needed to sort these six samples into three groups based on their odor quality similarity and then rank the odor intensity of the samples within the same group. Only the subjects who answered both parts correctly, i.e., gathered the samples with the same odor quality into a group and then correctly ranked the odor intensity within the groups, were selected for the experiment. To further test subjects’ scaling abilities, we provided six pictures proposed by Meilgaard et al. [4]. These pictures had different shadowed areas, and subjects had to evaluate the approximate area using a linear scale. The values given by subjects were compared to the correct values, and only subjects who gave substantially incorrect ratings were not selected.

All subjects provided informed consent in line with the Helsinki Declaration, and six subjects quit after the training session, leaving 60 subjects (41 female) to participate in the experiment. Before the main experiment, subjects participated in 2 training sessions that aimed to provide standards for intensity scale use (see below). During the main experiment, not all the subjects evaluated all the samples (hereafter called trials because each trial included 3 odorized vials); for a given sample, 30 subjects performed the evaluation. Trials were randomly assigned to the subjects, who participated in a minimum of 3 and a maximum of 15 sensory sessions, with a maximum of 3 sessions occurring per week. During a session, participants evaluated 8 to 10 trials. Subjects were paid for their participation.

### 2.2 Stimuli

Odor-active compounds occurring in natural products were the focus. To select these odor-active compounds, we included the 226 key food odorants (KFOs) identified in Dunkel et al. [3] and added 548 different odor-active compounds collected in the Flavornet database (http://www.flavornet.org/). Among the total of 774 compounds, we finally selected 72 (Table 1) that covered the odorant physicochemical space [7] and were easily available from providers. The idea of physicochemical space has been explained by Snitz, Yablonka, Weiss, Frumin, Khan, & Sobel, [6] and used by the same group in [7]. The so-called “physicochemical odor space” is based on the fact that odorants can be described by a large set of structural and physicochemical descriptors. It is thus possible to map large set of odorants using these descriptors that measure various properties and to obtain a “space” of odorants based on their physicochemical properties. Applied to our study, we considered 226 key food odorants (KFOs) identified in [3] and 548 different odorants collected in the Flavornet database (http://www.flavornet.org/). For this set of 774 odorants we obtained circa 4000 physicochemical descriptors using the Dragon® software (Talete, Milan, Italy). We mapped the set of odorants using the physicochemical descriptors and we selected 72 odorants that cover the whole map. Doing this, we aimed to cover a large range of structural and physicochemical properties, but using a limited set of odorants.

**Table 1:** Information and Final Concentration of Odorants Used in Each Trial.

CAS.	Odorant	Odor	Concentration (mg/mL)	Solvent	Purity	Trial number*
4180–23–8	1‑methoxy-4-[(*E*)‑*prop*-1-*en*-1-*yl*]benzene(trans-anethol)	anise	4.40	mineral oil	≥99%	35, 84, 90
100–52–7	benzaldehyde	almond	3.82	1,2-propanediol	99%	33, 135
7452–79–1	ethyl 2-methylbutyrate	apple	3.99	1,2-propanediol	99%	***5***, 16, ***22, 32, 36***, 58, ***59, 60***, 61, ***62, 63, 64***, 67, ***72***, 87, 92, ***94***, 95, 100, 104, 105, ***119, 134, 145***, 151, ***202, 215***, 219, 222
6378–65–0	hexyl hexanoate	apple peel	7.41	1,2-propanediol	>98%	18, 133, 171, 181
104–67–6	*γ*-undecalactone	apricot	33.5	1,2-propanediol	99%	49, 118, 125, 162, 174, 189
123–35–3	myrcene	balsamic	1.62	mineral oil	≥97%	38, 44, 45, ***60***, 82, ***94***, 103, 106, 107, ***116***, 136, 137, 205, ***210***, 218
431–03–8	diacetyl	butter	9.89	1,2-propanediol	99%	***5, 32***, 169, 194
513–86–0	acetoin	butter	25.2	water	≥97%	68, 146
3658–80–8	dimethyl trisulfide	cabbage	0.000805	1,2-propanediol	≥97%	65
96–48–0	*γ*-butyrolactone	caramel	143	1,2-propanediol	≥97%	79
103–36–6	ethyl cinnamate	cinnamon	8.25	1,2-propanediol	98%	30, 82, 88, 177, 178, 187, 193
4630–07–3	valencene	citrus	4.91	mineral oil	≥65%	***36***, 37, ***71***, 77, ***124***, 126, ***130***, 195, ***201, 202***, 203, 209, 216
99–87–6	*p*-cymene	citrus	3.27	mineral oil	≥97%	2, 7, 11, ***13, 14, 15***, 16, 17, 18, ***25, 28***, 43, 46, ***116***, 200, 208, 209, ***210, 211***, 214
97–53–0	eugenol	clove	0.467	1,2-propanediol	99%	21, 56, 80, ***86, 175***, 176, 185, 191, 217
105–21–5	*γ*-heptalactone	coconut	4.30	1,2-propanediol	≥98%	4, 8, ***14***, 20, ***26, 28, 29***, 30, 51, 52, 53, ***54, 62***, 85, 98, 108, 109, 110, ***114***, 118, ***119***, 120, 144, 149, 150
3268–49–3	methional	cooked potato	0.0680	1,2-propanediol	≥97%	4, 156, 157, ***163***, 167, ***182***, 197
695–06–7	4-hexanolide	coumarin	3.43	1,2-propanediol	≥97%	58
13623–11–5	trimethylthiazole	earth	1.01	1,2-propanediol	≥98%	100, 128
106–33–2	ethyl laurate	fat	97.9	1,2-propanediol	99%	19
111–13–7	2-octanone	fat	1.11	1,2-propanediol	≥97%	40, 81, 121, 197, 203, 206, 217
111–70–6	1-heptanol	fat	11.8	1,2-propanediol	≥99%	12, 41, 56, 144
124–13–0	octanal	fat	2.44	1,2-propanediol	99%	47, 57
106–25–2	nerol oxide	flower	2.66	mineral oil	≥97%	53, 133, 170, 180
140–11–4	benzyl acetate	flower	8.08	1,2-propanediol	99%	39, 83, 89
78–70–6	linalool	flower	6.12	1,2-propanediol	≥80%	21, 123, ***163***, 172, ***182***, 206, 214, 220
551–93–9	*o*-aminoacetophenone	foxy	49.8	1,2-propanediol	98%	97, 136
101–97–3	ethyl phenylacetate	fruit	1.18	1,2-propanediol	99%	27, 55, 57, 81, 87, 176, 177, 186, 192, 218
105–37–3	ethyl propionate	fruit	1.30	1,2-propanediol	99%	51
105–54–4	ethyl butyrate	fruit	8.14	1,2-propanediol	99%	33, 121, 168, 183, 198
105–57–7	diethyl acetal	fruit	2.00	1,2-propanediol	99%	41, 45, 49, ***63***, 70, ***71, 72***, 73, 90, 111, ***112, 127, 130***, 139, 148, 150, 153
106–32–1	ethyl octanoate	fruit	8.47	1,2-propanediol	≥99%	83, 117, 170, 185, 212
107–87–9	2-pentanone	fruit	1.66	1,2-propanediol	98%	31
108–64–5	ethyl 3-methylbutanoate	fruit	0.127	1,2-propanediol	98%	74, 91, 155, 160, 161, 165, 180, 195
2305–05–7	*γ*-dodecalactone	fruit	22.4	1,2-propanediol	≥97%	23, 174, 184
539–82–2	ethyl valerate	fruit	7.18	1,2-propanediol	≥98%	2, 123, 155, 156, 162, 166, 181, 196
137–32–6	2-methyl-1-butanol	fusel oil	7.20	1,2-propanediol	≥99%	43
66–25–1	hexanal	grass	1.26	1,2-propanediol	98%	10, 11, 38, 198, 204, ***207, 213***
928–96–1	(*Z*)−3-hexenol	grass	1.44	1,2-propanediol	98%	9, 37, 42, ***59, 145***, 161, 199, 204, 205
123–72–8	butanal	green	10.8	1,2-propanediol	≥97%	34
103–45–7	*β*-phenethyl acetate	honey	9.92	1,2-propanediol	99%	***15***, 79, 85, ***86, 175***, 190, ***211***, 216
122–78–1	phenylethanal	honey	2.32	1,2-propanediol	≥90%	88, 120, 151, 152
96–17–3	2-methylbutanal	malt	3.57	1,2-propanediol	95%	76, 108
106–44–5	*p*-cresol	medicine	5.53	1,2-propanediol	≥97%	40, 154
470–82–6	1,8-cineole	mint	4.62	1,2-propanediol	≥97%	1, 7, 8, 9, 24, 68, ***75, 124***, 128, ***131***, 196, ***201***, 212
543–49–7	2-heptanol	mushroom	1.74	1,2-propanediol	≥97%	39, 105, 110, 143
13327–56–5	ethyl 3-(methylsulfanyl)propan oate	onion	0.373	1,2-propanediol	>99%	34, 42, 46, ***50, 54, 64, 69***, 73, 74, ***75, 113, 114***, 115, ***131, 132, 134***, 135, 137, 140, 154
111–11–5	methyl octanoate	orange	2.73	1,2-propanediol	99%	61
706–14–9	*γ*-decalactone	peach	7.36	1,2-propanediol	≥98%	78, 140, 173, 188, ***207, 213***
123–86–4	butyl acetate	pear	2.31	1,2-propanediol	99%	6, 158, 159, 167, 192
505–10–2	methionol	potato	0.593	1,2-propanediol	98%	55, 166, 191
110–62–3	pentanal	pungent	12.0	1,2-propanediol	≥95%	31, 66
693–95–8	4-methylthiazole	roasted meat	0.385	1,2-propanediol	≥98%	98, 101
104–76–7	2-ethylhexanol	rose	9.46	1,2-propanediol	≥99%	10, 78, 107, 141
105–87–3	geranyl acetate	rose	10.4	1,2-propanediol	98%	76, 138, 171, 186
106–22–9	citronellol	rose	3.65	1,2-propanediol	≥97%	48, 77, 139, 172, 187
106–24–1	geraniol	rose	4.79	1,2-propanediol	98%	***22***, 142, 164, 173, 183, 208, ***215***
4410–99–5	phenylethylthiol	rubber	0.279	1,2-propanediol	≥97%	6, 12, 17, 23, 66, 67, ***69***, 89, ***96***, 102, 103, ***112***, 122, ***127, 129, 132***, 147, 149, 152
3391–86–4	1-octen-3-ol	soap	1.48	1,2-propanediol	98%	52, 102, 142
821–55–6	2-nonanone	soap	3.05	1,2-propanediol	≥97%	48
99–48–9	carveol	spearmint	9.68	1,2-propanediol	97%	35, 80, ***96***, 104, 106, 109, 125, ***129***
2785–89–9	4-ethylguaiacol	spice	3.58	1,2-propanediol	98%	99, 179, 189, 219
97–54–1	isoeugenol	spice	3.33	1,2-propanediol	≥97%	91, 115, 178, 179, 188, 194
88–15–3	acetylthiophene	sulfur	1.62	1,2-propanediol	≥98%	99, 111
503–74–2	isovaleric acid	sweat	79.0	1,2-propanediol	98%	3
112–44–7	undecanaldehyde	sweet	0.357	1,2-propanediol	≥97%	20
123–11–5	*p*-anisaldehyde	sweet	4.24	1,2-propanediol	≥99%	95
97–62–1	ethyl isobutyrate	sweet	4.49	1,2-propanediol	99%	1, 70, 159, 160, 168, 193, 200
18640–74–9	isobutyl thiazole	tomato leaf	0.315	1,2-propanediol	99%	101, 138
51755–83–0	3-mercaptohexanol	tropical fruit	0.593	1,2-propanediol	98%	220, 221, 222
121–33–5	vanillin	vanilla	0.786	1,2-propanediol	98%	65, 93, 122, 153
123–51–3	3-methyl-1-butanol	whisky	2.06	1,2-propanediol	≥99%	47, 141, 157, 158, 164, 165, 169, 184, 190, 199, 221
123–25–1	diethyl succinate	wine	388	1,2-propanediol	98%	3, ***13***, 19, 24, ***25, 26***, 27, ***29***, 44, ***50***, 84, 92, 93, 97, ***113***, 117, 126, 143, 146, 147, 148

⁎ The 24 trial numbers marked with bold fonts are duplicate trials.

Most odorants were purchased from Sigma-Aldrich China Co. (Shanghai, China) in the highest available purity, except for p-anisaldehyde (obtained from Fluka) and 3-mercaptohexanol (obtained from ACROS Organics). Ultimately, 198 different binary odor mixtures (Table 1), plus 24 duplicated binary odor mixtures, made from the 72 odorants were designed for the experiments based on their odor characteristics.

All odorants were diluted with odorless solvents which were 1,2-propanediol, or mineral oil or deionized distilled water depending on odorant solubility. To avoid large differences in intensity and to keep it in a narrow range for all samples, odorants were first diluted to a point approximately equal to the odor intensity of ethyl 2-methylbutyrate at a concentration of 3.9 g/L, as estimated by experienced lab members. Then, we prepared a set of solutions of odorants varying around the obtained concentration. These solutions were presented to 6 subjects who did not participate in the main experiment and who were instructed to provide a number between 0 and 7 reflecting the solution odor intensity. For each odorant, the final concentration (Table 1) was set after the rough isointensity and was defined following the procedure described in Weiss et al. [7].

### 2.3 Sample preparation

To prevent the formation of novel chemicals in the mixtures, odorants were not mixed in the liquid phase. For the unmixed odor samples, 200 µL of diluted stimulus was poured onto a 0.1 g cotton ball and placed in a 20 mL brown glass bottle with black screw cap. For the binary mixtures, 200 µL of each stimulus was poured onto separate sides of the 0.1 g cotton ball, such that the two odorants’ vapors alone mixed in the glass bottle headspace. All of the stimuli were fully absorbed by the cotton ball. All samples were prepared one day before the sensory session and stored at room temperature (24 °C).

### 2.4 General procedures

Before the formal experiment, we began with two training sessions. The first session determined the standard odor references to be used in the experiment. Ethyl 2-methylbutyrate and linalool were selected as reference odor-active compounds because the majority of panelists did not object to sniff it frequently, and because their corresponding odors (fruity-green-apple and floral-citrus-lavender respectively) were rather familiar to the participants, which might have helped them to memorize. To determine the standard intensity of these references, we gave participants ethyl 2-methylbutyrate (1.8 g/L) and linalool (10.7 g/L) and asked them to rate the intensity of these two samples. We asked them to evaluate ethyl 2-methylbutyrate first, and then, they need to evaluate the intensity of linalool by comparing the intensity of linalool with the intensity of ethyl 2-methylbutyrate. If the intensity of linalool smelled twice as strong as ethyl 2-methylbutyrate, its intensity was marked twice the distance from zero as the position of ethyl 2-methylbutyrate. The standard intensity was obtained by calculating the mean value of these ratings across all subjects. The intensities of standard I (ethyl 2-methylbutyrate) and standard II (linalool) were finally anchored as 3.0 and 7.0, respectively. The second session introduced the odor evaluation procedures. During this session, the two standards were provided to the subjects, and they were told that they needed to rate the perceived intensity of the samples presented during the formal sessions using the anchor intensities of the two standards.

In the formal sessions, a total of 222 trials, among which 24 were duplicated trials, were evaluated. Each session in the formal experiment comprised 14 to 15 trials, and each trial included three stimuli: two stimuli were single odorants, and the third stimulus was a binary mixture of these odorants. In each trial, all the unmixed odor samples were coded by three random digits, and the binary mixture sample was coded by its trial number. The binary mixture was always presented first and the order of presentation of the two unmixed odors was counterbalanced for each trial, and each trial was presented to the subjects in a random order. Subjects were given a rest of 45 s between each stimulus. Each trial was presented to subjects in a random order, and one trial was evaluated by a maximum of 30 subjects.

Each session included two parts (Fig. 1). The first part consisted of a hedonic evaluation, and the other part consisted of intensity evaluations. During the hedonic evaluation, subjects had to mark off distance on a visual analog scale 100-mm in length (Fig. 1). For the intensity evaluations, an adjusted explicit anchoring scale with markers of two standards that were determined in the training session was utilized in the odor intensity evaluation. This kind of anchoring scale, with the reference standards used in this study, was aimed at familiarizing the panelists with the scale in a similar way across the range of intensity. This scale has been employed in texture analysis and might generate more reliable sensory data by reducing the variability among the panelists [5]. To rate the intensity, the subjects had to mark off a distance on the visual analog scale according to the two perceptual anchors. They were instructed that if the test stimulus smelled half as strong as the standard, its intensity should be marked half the distance, while if the stimulus smelled twice as strong as the standard, its intensity should be marked twice the distance from zero as the standard position. They should consider the two references to rate a given sample intensity (Fig. 1). The two standards were presented in the first two sessions to help the subjects rate the odor intensity. To evaluate the intensity (IAmix, IBmix) of the two components’ odor perceived within the mixture, the subjects had to indicate if they perceived odor A and/or odor B in the mixture and then had to evaluate the intensity (IAmix, IBmix) of the two components’ odor perceived within the mixture in comparison to the intensity of the unmixed components. If the subjects perceived the intensity (IAmix, IBmix) of the two components’ odor in the mixture to exceed the maximum of the scale, they were told to indicate it using a note. In this case, a maximum value of 11 for intensity was attributed instead of a maximum value of 10, which corresponds to the maximum value of the scale. At the end of the intensity evaluations, subjects were also asked to answer whether they could perceive a new odor in the mixture (data not shown).

## Ethics Statement

To conduct our experiments with human volunteers, we strictly followed the regulations applicable at the time the research was conducted at the Jiangnan University. Therefore, all participants were provided informed consent in line with the Declaration of Helsinki regarding human experimentation. They signed the agreement and volunteered to participate in the experiment and were paid for their participation.

## CRediT Author Statement

**Yue Ma:** Conceptualization, Methodology, Investigation, Data curation, Formal analysis, Writing - original draft; **Ke Tang:** Methodology, Conceptualization, Resources, Project administration, Writing - review & editing; **Yan Xu:** Project administration, Supervision, Resources, Funding acquisition, Writing - review & editing; **Thierry Thomas-Danguin:** Conceptualization, Formal analysis, Supervision, Writing - review & editing.

## Funding

This work was supported by the 10.13039/501100012167National Key R&D Program (2016YFD0400504), 10.13039/100013097National First-class Discipline Program of Light Industry Technology and Engineering (LITE 2018–12), 10.13039/501100004543China Scholarship Council (201,806,790,033), and Postgraduate Research & Practice Innovation Program of Jiangsu Provence (KYCX18_1788).

## Declaration of Competing Interest

The authors declare that they have no known competing financial interests or personal relationships which have or could be perceived to have influenced the work reported in this article.
